# Genome-Wide Identification, Expression, and Functional Analysis of UDP-Glucose Dehydrogenase Family Genes in *Rhus chinensis*

**DOI:** 10.3390/genes17060705

**Published:** 2026-06-18

**Authors:** Guang Ba, Ke Hu, Youyang Wang, Yiyu Tang, Chengxiong Liu, Wen Liu

**Affiliations:** 1 Biotechnology Research Center, Key Laboratory of Three Gorges Regional Plant Genetics & Germplasm Enhancement (CTGU), College of Biological and Pharmaceutical Sciences, China Three Gorges University, Yichang 443002, China; bg2062373423@126.com (G.B.); 13094170426@163.com (K.H.); wangyouyang0506@126.com (Y.W.); 18017746408@163.com (Y.T.); 2 Hubei Key Laboratory of Natural Products Research and Development, College of Biological and Pharmaceutical Sciences, China Three Gorges University, Yichang 443002, China; liuchengxiong666@126.com

**Keywords:** *R. chinensis*, gallotannins, UDP-glucose dehydrogenase (UGD), gene expression, biosynthesis, enzyme activity

## Abstract

**Background**: Uridine diphosphate glucose (UDP-Glc) is one of the key substrates for the biosynthesis of gallotannins in plants. UDP-glucose dehydrogenase (UGD) catalyzes the irreversible oxidation of UDP-Glc to UDP-glucuronic acid (UDP-GlcA), thus affecting the biosynthesis and accumulation of gallotannins in the Chinese gallnut. **Methods and Results**: In this study, we identified three members of the *RcUGD* family from the *Rhus chinensis* genome. Protein sequence alignment revealed that all three RcUGDs possess the conserved NAD^+^ coenzyme binding motif GAGYVGG and the catalytic motif GFGGSCFQKDIL. qRT-PCR analysis revealed that the expression levels of *RcUGD3* in stem and root tissues were respectively 10-fold and 13-fold greater than that in the leaves, in which gallotannin accumulation was higher. *RcUGD3* expression level declined by 63% during early (24 d) gallnut development, suggesting an inverse relationship between *RcUGD3* expression level and gallotannin biosynthesis. In addition, subcellular localization analysis using the tobacco transient transformation system showed that RcUGD proteins are broadly distributed throughout the cell. Moreover, an in vitro enzyme activity assay indicated that the recombinant RcUGD3 protein catalyzed UDP-Glc to produce UDP-GlcA as shown by HPLC. Taken together, our results suggested that RcUGD3 protein is responsible for UDP-Glc degradation and probably plays a regulatory role in gallotannin biosynthesis in the Chinese gallnut. **Conclusions**: This study lays a foundation for further elucidating the function and expression regulation mechanism of the *RcUGD* gene family and provides new insights for the super-accumulation mechanisms of gallotannins in Chinese gallnuts.

## 1. Introduction

UDP-Glc, as one of the most important reactive sugars in plant cells, serves as a core “metabolic hub molecule” connecting primary metabolism and secondary metabolism [1]. As a vital upstream metabolite, it participates in fundamental primary metabolic processes and carbon allocation in plants [2,3,4,5]. UDP-GlcA is synthesized from UDP-Glc via the catalysis of UDP-glucose dehydrogenase (UGD) and exhibits diverse metabolic functions. It provides critical substrates for the glycosylation modification of secondary metabolites, including flavonoid glycosides and lignin, and participates in the regulation of secondary metabolite accumulation. Moreover, this metabolite contributes to plant adaptation to biotic and abiotic stress conditions [6]. The oxidative dehydrogenation of UDP-Glc to produce UDP-GlcA constitutes a key directional shunt in the UDP-Glc metabolic cascade. The regulatory balance of this pathway directly modulates the metabolic resource distribution between primary and secondary metabolism in plants.

UGD catalyzes the oxidation of UDP-Glc to produce UDP-GlcA. Research on UGDs was initiated in prokaryotes [7]. Subsequently, identification and functional characterization of the *UGD* gene family have been reported in numerous plant species such as *Glycine max* [8], *Arabidopsis thaliana* [9], *Nicotiana tabacum* [10], and *Populus tremula x tremuloides* [11]. In the model plant *A. thaliana*, the *AtUGD* family members were shown to be closely related to cell wall synthesis and stress response [12]. The double mutant *ugd2ugd3* exhibited phenotypes such as defective seedling development, slow growth, and dwarfism, directly demonstrating the necessity of UGD enzymes for maintaining cell wall integrity [13]. Overexpressing *LgUGDH* in *A. thaliana* significantly increased the content of soluble sugars and hemicellulose, enhancing both growth rate and cold tolerance [14]. Similarly, overexpressing *PeUGDH1* in *A. thaliana* also elevated the content of hemicellulose and soluble sugars, further indicating the conserved function of *UGD* in plant sugar metabolism and cell wall biosynthesis [6]. On the other hand, expressing the antisense *GbUGD6* gene from *Gossypium barbadense* disrupted the structural integrity of the cell wall, leading to decreased biomass accumulation and morphological abnormalities in *A. thaliana*, once again highlighting the important function of *UGD* genes in plant growth and development [15].

*R. chinensis*, a species of economic forest trees in China, belongs to the genus *Rhus* in the family Anacardiaceae. The Chinese gallnut, a traditional medicine in China, is formed on the leaves of *R. chinensis* induced by the parasitism of the Chinese gallnut aphid [16]. Gallotannins are the main bioactive components in the Chinese gallnut. As important hydrolyzable tannins, gallotannins are widely used in medicine, food preservation, ecological environmental protection, and leather tanning [17,18]. Previous research has indicated that, during the development of the gallnut, gallotannins are also highly accumulated with a top content of 70% of total dry weight [19]. The features of the formation of the insect gall and high accumulation of gallotannins let *R. chinensis* draw the attention of researchers. Recently, two groups reported the genome database of *R. chinensis* [20,21], which facilitates further functional gene isolation and exploration of molecular mechanisms underlying special phenotypes in *R. chinensis*.

The biosynthesis of gallotannins in plants requires the substrates of gallic acid and glucose, and UDP-Glc is the main sugar donor [22]. Thus, the metabolic flow of UDP-Glc in *R. chinensis* is unique: it not only needs to enter the UDP-GlcA-dependent pathway (supporting cell wall synthesis and conventional secondary metabolism) catalyzed by UGD but also serves as a key starting material, combining with gallic acid under the catalysis of UDP-glucosyltransferase (UGT) to generate 1-O-galloylglucose (β-galloylglucose), which is the initial key step in the synthesis of gallotannins [22,23]. Subsequently, this intermediate product gradually extends into complex tannic acid molecules through multiple acyltransferase reactions [22,24]. This indicates that the “diverging reaction” catalyzed by the UGD enzyme in *R. chinensis* may have a potential “metabolic competition” relationship with the synthesis of gallotannins: when UGD activity is enhanced, more UDP-Glc will be directed to the UDP-GlcA pathway, which may indirectly reduce the precursor supply for gallotannin biosynthesis; conversely, if UGD activity is temporally and spatially downregulated, it may reserve more UDP-Glc for gallotannin synthesis, thereby affecting the developmental quality of the Chinese gallnuts and the accumulation of gallotannins. Nevertheless, the molecular features and regulatory expression patterns of *RcUGD* genes have not yet been systematically characterized. This knowledge gap severely restricts our insight into secondary metabolic pathways in this species and impedes the development of its functional genetic resources.

In this study, we performed a genome-wide identification of the *RcUGD* gene family in *R. chinensis*, followed by systematic analyses of their phylogenetic relationships, tissue-specific expression patterns, and enzyme activity. Our study will elucidate the molecular characteristics and functional basis of the *RcUGD* gene family in *R. chinensis* and provide a theoretical basis for subsequently optimizing the synthesis of gallotannins by regulating UGD activity.

## 2. Materials and Methods

### 2.1. Plant Materials and Growth Conditions

The seeds and the horned galls at different developmental stages were collected from cultivated *R. chinensis* tree species in the mountainous area with an altitude of 1000 m in Wufeng, Hubei province, China (111°15′ E, 30°19′ N). For the horned galls, the plant samples were gathered after removing the gall-forming aphids (*Schlechtendalia chinensis*) inside, frozen in liquid nitrogen immediately and kept at −80 °C until use. The day on which cultivated gall-forming aphids were placed artificially onto plants was designated as Day 0. The different plant tissues for gene expression analysis were collected from 1-month-old seedlings germinated from mature seeds. The seedlings were grown in a growth chamber kept at 23 °C under a 16 h light/8 h dark cycle at a light intensity of 80 mmol photons m^−2^ s^−1^.

### 2.2. Identification and Physicochemical Analysis of the RcUGD Genes

The *RcUGD* family genes in *R. chinensis* were identified based on the genome data [20,21]. Briefly, the protein sequences of *AtUGD* genes were downloaded from the TAIR website (https://www.arabidopsis.org/) (accessed on 3 June 2025) to serve as queries. The *RcUGD* homologous genes were identified by BLAST using the TBtools software (v2.485), with an E-value threshold set to 1 × 10^−5^ [25]. Then, the key domains of their coding proteins were predicted by the online database Pfam (http://pfam.xfam.org/) (accessed on 3 June 2025) [26] and SMART (https://smart.embl.de/) (accessed on 3 June 2025) [27]. And their physicochemical properties were analyzed by the Expasy database (https://www.expasy.org/) (accessed on 4 June 2025) [28]. Meanwhile, the transmembrane domain and the secondary structures of these proteins were predicted by the TMHMM database (https://services.healthtech.dtu.dk/services/TMHMM-2.0/) (accessed on 4 June 2025) and the SOPMA database (https://npsa.lyon.inserm.fr/cgi-bin/npsa_automat.pl?page=/NPSA/npsa_sopma.html) (accessed on 4 June 2025), respectively.

### 2.3. Evolutionary Characteristics and Chromosomal Localization of the RcUGD Genes

Multiple sequence alignment of the RcUGD protein sequences was performed using ClustalW integrated into MEGA 11.0 [29]. Then, an unrooted phylogenetic tree was constructed by the neighbor-joining method with 1000 bootstrap iterations under the p-distance model [30]. The chromosomal localization of *RcUGD* genes was visualized by the TBtools software. The collinearity analysis of *RcUGD* genes was conducted by MCScanX software and visualized by the TBtools software.

### 2.4. Analysis of RcUGD Protein Sequences and Cis-Acting Elements in the Promoter Regions

The homologous sequence alignment analysis of RcUGD proteins was performed by the DNAMAN 7.0 software. The conserved motifs of RcUGD proteins were analyzed by the MEME database (https://meme-suite.org/meme/tools/meme) (accessed on 10 June 2025). The maximum number of identified motifs was set to 10, and the other parameters were kept at default settings. The exon–intron structures of *RcUGD* genes were visualized by the GSDS 2.0 database (https://gsds.gao-lab.org/Gsds_help.php) (accessed on 10 June 2025). The 2 kb upstream promoter sequences of *RcUGD* genes were isolated from the genome data of *R. chinensis* using the TBtools software, analyzed by the PlantCARE website (https://bioinformatics.psb.ugent.be/webtools/plantcare/html/) (accessed on 10 June 2025), and finally visualized by the TBtools software.

### 2.5. RNA Extraction and qRT-PCR

The total RNA was isolated by a FastPure^®^ Universal Plant Total RNA Isolation Kit (Vazyme, Nanjing, China) according to the manufacturer’s instructions [31]. After digestion by DNase I (Vazyme, Nanjing, China), cDNA was obtained using the BeyoRT II First-stand cDNA Synthesis Kit (Beyotime, Shanghai, China). Then, qRT-PCR was conducted on a Bio-Rad CFX96 apparatus with TB Green^®^ Premix Ex TaqTM (Tli RNaseH Plus) (Takara, Beijing, China) [32]. PCR reaction was carried out in 96-well plates under a two-step procedure: 3 min incubation at 95 °C for complete denaturation, followed by 45 cycles of 95 °C for 15 s and 60 °C for 45 s, ended by a melt curve from 60 °C to 95 °C. *RcPP2A* (*serine/threonine protein phosphatase 2A*) was selected as an internal control, since its expression stability in *R. chinensis* has been validated by our previous studies. Gene relative expression level was calculated via the 2^−ΔΔCt^ method [33]. The specificity of the primers was verified by the melting curve, and PCR efficiency was set between 90% and 110%. At least three independent biological replicates and three technical repetitions were performed for each data. The primer sequences for qRT-PCR are listed in Table 1.

### 2.6. Subcellular Localization of RcUGD Proteins

Subcellular localization was performed by transiently expressing RcUGD proteins fused with green fluorescent protein (GFP) in tobacco leaves as previously reported [34]. The coding sequences of *RcUGD* genes were amplified by PCR and inserted in-frame into the binary vector pCAMBIA1300s-GFP via an in-fusion cloning method. After being transformed into *Agrobacterium tumefaciens* GV3101 (WEIDI, AC1001), the strains were syringe-infiltrated into *Nicotiana benthamiana* leaves. The transformed plants were kept in darkness for one day, followed by incubation in normal conditions for two days [35]. Thereafter, the subcellular localization of RcUGDs was analyzed by detecting the fluorescent signals with a confocal laser-scanning microscope (A1R^+^, Nikon, Tokyo, Japan). Control was conducted by transforming the empty vector into *N. benthamiana* leaves. At least three independent biological replicates were performed for each protein.

### 2.7. Purification of Recombinant Protein and In Vitro Enzyme Activity Assay

For prokaryotic protein purification, recombinant vectors were constructed by inserting the coding sequences of *RcUGDs* into the pET28a vector. After being transformed into the *Escherichia coli* BL21(DE3) competent cells (Solarbio, Beijing, China), recombinant strains were then induced by 1 mM isopropyl-β-D-thiogalactoside (IPTG, Beyotime, ST098) at 25 °C for 12 h. Subsequently, recombinant proteins were purified by the His-tag Protein Purification Kit (Beyotime, Shanghai, China) according to the manufacturer’s instruction. Protein samples collected from bacterial strains before and after 12 h of IPTG induction, cell lysate, flow-through fraction and purified protein were mixed with 5× SDS loading buffer (Beyotime, P0295) at an appropriate ratio, followed by boiling in a water bath for 5 min for denaturation. The prepared samples were loaded onto a 12% SDS–polyacrylamide gel (Vazyme, Nanjing, China) and electrophoresed at a constant voltage of 100 V. After electrophoresis, the gel was stained with Coomassie Brilliant Blue R-250 (Vazyme, Nanjing, China) for 30 min at room temperature and then destained until distinct protein bands were observed.

For in vitro protein activity analysis, a 100 μL reaction system was set up containing 10 μg purified recombinant proteins, 50 mM Tris-HCl at pH 7.5 (Solarbio, Beijing, China), 1 mM UDP-Glc (Yuanye, Shanghai, China), and 2 mM NAD^+^ (Aladdin, Shanghai, China). The reaction mixture without proteins was set as a negative control. The mixture was vortexed and incubated at 30 °C for 30 min, followed by mixing 10 µL trichloroacetic acid and 100 µL methanol to stop the reaction. After centrifuging at 15,000 rpm for 10 min at 4 °C and filtering the supernatant through a 0.22 μm microporous membrane, the reaction solution was separated on a Shimadzu LC-2030C HPLC with a COSMOSIL 5C18-MS-II column (250 × 4.6 mm, particle size of 5 μm) under 35 °C. The mobile phase consisted of 10 mM KH_2_PO_4_, pH 6.0 (eluent A) (Macklin, Shanghai, China) and acetonitrile (eluent B) (TEDIA, purity ≥ 99.9%, HPLC grade, Anqing, China) with a flow rate of 0.8 mL/min. The linear gradient was 7% eluent B (0–30 min). The in vitro enzyme activity assay was performed three times.

### 2.8. Statistical Analysis

For each experiment, at least three biological repetitions and three technical repetitions were carried out. For multiple comparisons, the Graphpad 9.0 software was used to confirm the normality and homogeneity of variances of the data and to perform one-way ANOVA; then the significance of differences between multiple samples was determined by the Waller–Duncan test (*p* < 0.05). For qRT-PCR, the relative expression levels were analyzed, and comparisons were performed between different organs or between different developmental stages.

## 3. Results

### 3.1. Identification and Physicochemical Analysis of the RcUGD Genes

Here, we identified three *UGD* family members from the genome of *R. chinensis*, which were named *RcUGD1*, *RcUGD2*, and *RcUGD3*. The main features of their coding proteins were quite similar, including molecular weight, pI, instability index, aliphatic index, CRAVY (Table 2), and secondary structures (Table 3). All three RcUGD proteins were slightly acidic, suggesting that these proteins carry a net negative charge at physiological pH, a property that may facilitate interactions with positively charged substrates such as NAD^+^. The instability index of RcUGD proteins ranged from 23.85 to 25.44, indicating that they were all stable proteins. Meanwhile, the RcUGD proteins exhibited a high aliphatic index, suggesting good protein stability under different environments. The secondary structure prediction revealed that α-helices and random coils are the main components, with a small proportion of extended chains but no β-turns (Table 3). The protein property analysis confirmed that all three RcUGD proteins are canonical UGD family members based on MW, pI, stability, and hydrophilicity.

### 3.2. Phylogenetic Analysis of RcUGD Family Genes

**Figure 1:**
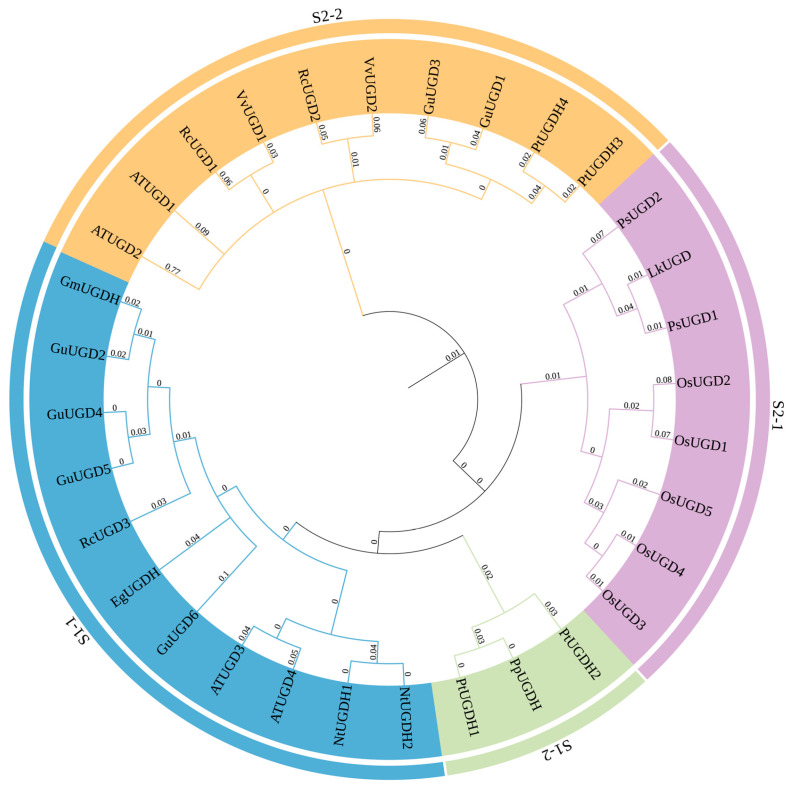
Phylogenetic analysis of the UGD gene family from *A. thaliana* (At), *G. max* (Gm), *Glycyrrhiza uralensis* (Gu), *R. chinensis* (Rc), *Eucalyptus grandis* (Eg), *N. tabacum* (Nt), *P. tremula* × *P. tremuloides* (Pp), *Oryza sativa* (Os), *Picea sitchensis* (Ps), *Larix kaempferi* (Lk), *Vitis vinifera* (Vv), *P. tomentosa* (Pt). The subfamilies are color-coded as follows: S1-1 (blue), S1-2 (green), S2-1 (purple), and S2-2 (orange).

To gain insight into the evolutionary relationships of *RcUGD* homologous genes, an unrooted neighbor-joining (NJ) phylogenetic tree was constructed by comparing the protein sequences of known UGD proteins from *A. thaliana*, *G. max*, and *Populus tomentosa* (Figure 1). We found that UGD homologous proteins could be divided into four major clades, as previously reported [36]. RcUGD1 and RcUGD2 were distributed into the S2-2 clade, while RcUGD3 was located in the S1-1 clade.

### 3.3. Chromosomal Mapping and Collinearity Analysis of RcUGD Family Genes

**Figure 2:**
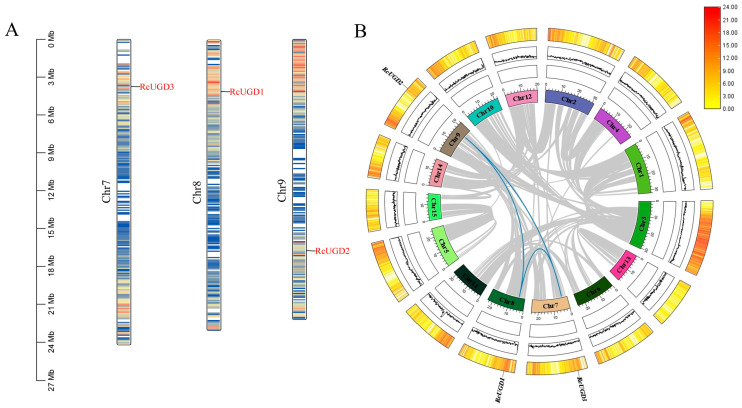
Chromosomal localization and collinearity analysis of *RcUGD* genes. (**A**) Chromosomal mapping of *RcUGDs*; (**B**) collinearity analysis.

Chromosome localization analysis suggested that *RcUGD3* is located in the approximately 3–6 Mb region of chromosome 7; *RcUGD1* is distributed in the upper segments of chromosome 8; and *RcUGD2* is localized in the 15–18 Mb interval of chromosome 9 (Figure 2A). The discrete distribution of different members on chromosomes indicates that the *RcUGD* genes may have undergone differentiation through evolutionary events such as chromosomal translocation, providing a localization basis for subsequent exploration of the relationship between gene functional differentiation and chromosomal evolution.

To explore the evolutionary origin and genome duplication characteristics of the *RcUGD* family members, a collinearity analysis was conducted using the MCScanX method. The results indicated three segmental duplication events involving three chromosomes (Figure 2B). *RcUGD1* and *RcUGD2*, *RcUGD1* and *RcUGD3*, and *RcUGD2* and *RcUGD3* are gene pairs on Chr8 and Chr9, Chr8 and Chr7, and Chr9 and Chr7, respectively. Extensive collinearity blocks (connected by gray lines) exist within the *R. chinensis* genome, suggesting that these regions may originate from genome duplication events. Furthermore, no direct tandem duplication relationships (i.e., closely adjacent duplications on the same chromosome) were found between *RcUGDs*, indicating that the amplification of this gene family in the *R. chinensis* genome may primarily rely on whole-genome duplication (WGD) or large-segment duplication events rather than on local duplications.

### 3.4. Sequence Alignment and Conserved Motif Analysis of RcUGD Proteins

**Figure 3:**
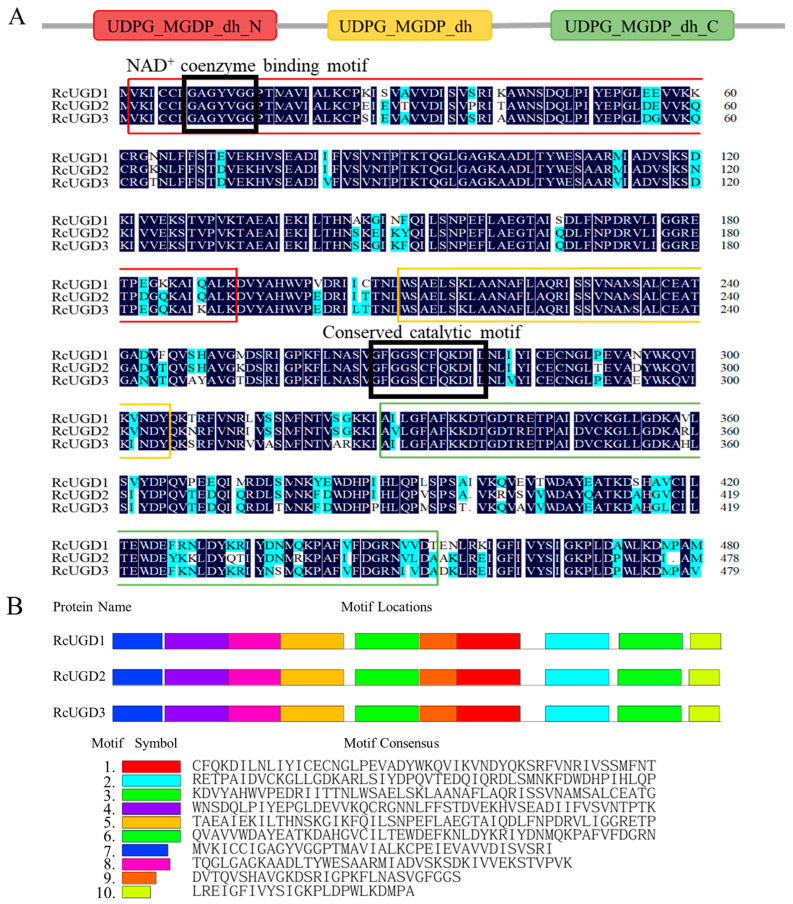
Sequence alignment and conserved motif analysis of RcUGD proteins. (**A**) Conserved domains of the RcUGD proteins; (**B**) analysis of conserved motifs in RcUGD proteins.

Multiple sequence alignment using DNAMAN 9.0 software revealed that all three RcUGD proteins possessed three typical conserved domains of the UGD family, including the N-terminal UDPG-MGDP-dh-N domain, the central UDPG-MGDP-dh core domain, and the C-terminal UDPG-MGDP-dh-C domain (Figure 3A). Additionally, an NAD^+^ coenzyme binding motif (GAGYVGG) and a catalytic motif (GFGGSCFQKDIL) were located in all three RcUGD proteins, which was consistent with previous findings [36].

Analysis of protein motifs indicated that 10 characteristic motifs (Motif 1–10) with similar distribution lay in three RcUGD proteins (Figure 3B). Further analysis revealed that Motif 7 corresponded to the core sequence of the UDPG-MGDP-dh-N domain (including conserved fragments such as MVKICCIGAGYVGGP); Motifs 3, 4, and 5 covered the key regions of the UDPG-MGDP-dh catalytic domain; and Motifs 2 and 6 matched the sequence of the UDPG-MGDP-dh-C domain. These results provide evidence for the conservation of this family of proteins at the motif level.

### 3.5. Analysis of the Gene Structure and Upstream Cis-Acting Elements of RcUGD Genes

**Figure 4:**
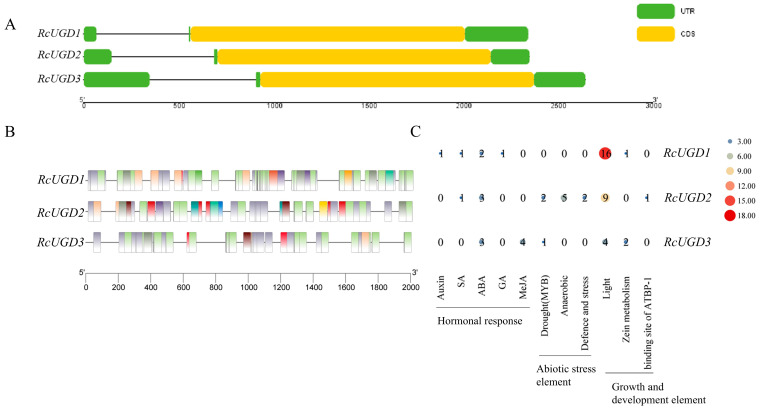
Analysis of the gene structure and upstream cis-acting elements of *RcUGD* genes. (**A**) Structural analysis of the *RcUGD* genes; (**B**) prediction of cis-acting elements in promoters; (**C**) analysis of the number of key elements.

To reveal the structural characteristics of the *RcUGD* genes, its exon/intron distributions were analyzed. We found that all three genes contained a continuous and intron-free coding sequence (Figure 4A), like other *UGD* homologous genes [6], while the differences mainly lay in the length of the UTR region, which affects the post-transcriptional regulation of the *RcUGD* genes (such as mRNA stability and translation efficiency).

To further explore the possible upstream regulatory network of *RcUGD* genes, we isolated their 2 kb promoter sequences and predicted the potential cis-acting elements using PlantCARE. Our results showed that various cis-acting elements were recognized in the promoter regions of the *RcUGD* genes, such as coenzyme response elements, multiple stress response elements, hormone response elements, and light response elements (Figure 4B). The variation in cis-acting elements in *RcUGD* promoters suggests that they may respond to different environmental and hormonal signals.

### 3.6. Subcellular Localization of RcUGD Proteins

**Figure 5:**
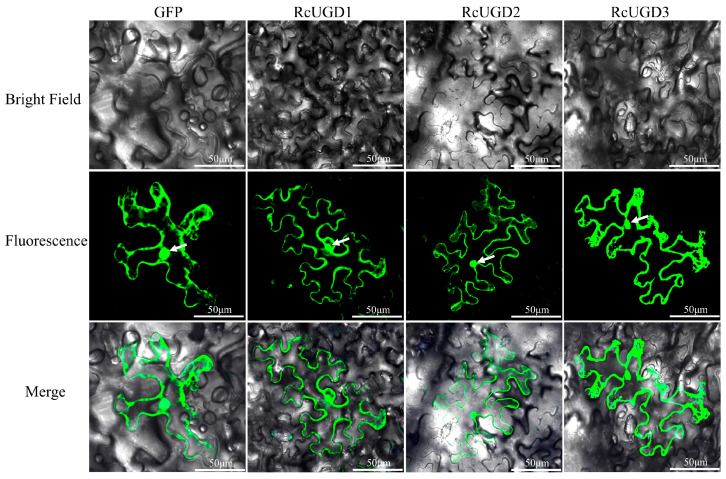
Subcellular localization of RcUGD proteins. Subcelluar localization of RcUGDs was observed in tobacco leaves by confocal microscopy. Merge stands for superimposed field. Fluorescence represents green fluorescence field. Bright Field represents bright field. The arrows point to the location of the cell nucleus. Bars = 50 μm.

To investigate the subcellular localization of RcUGD proteins, they were fused in-frame with GFP, transiently expressed in *N. benthamiana* leaves, and then visualized under confocal microscopy 3 days post-infiltration. As shown by GFP fluorescence, three RcUGD proteins were localized in both the cytosol and the nucleus (Figure 5).

### 3.7. Expression Patterns of RcUGD Genes

**Figure 6:**
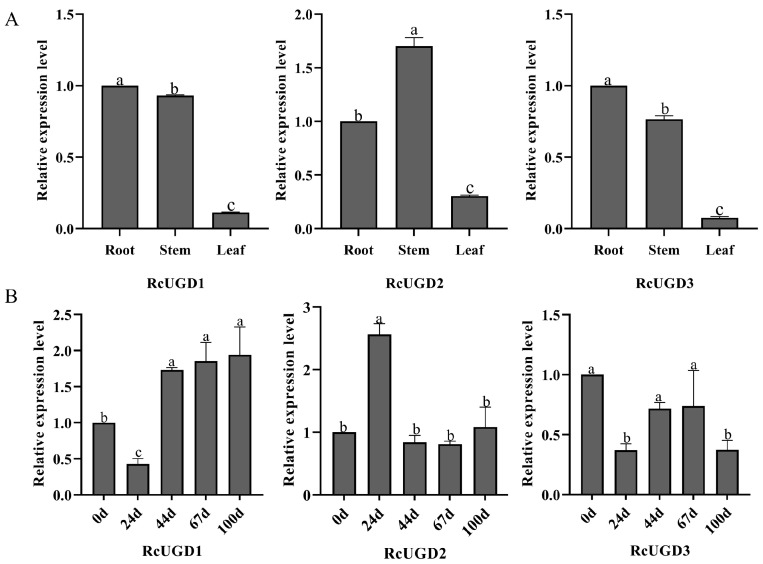
Expression patterns of *RcUGD* genes. (**A**) Expression levels of *RcUGD* genes in different organs (root, stem, leaf). (**B**) Expression levels of *RcUGD* genes in different developmental stages of the diploid. The bar charts show the standard deviation (SD) from three biological replicates. Error bars represent the standard deviation (SD) from three biological replicates. Different letters indicate significant differences between pairs.

To illustrate the expression profiles of *RcUGD* genes, their relative expression levels in different tissues in *R. chinensis* were analyzed by qRT-PCR. We found that, in leaves in which the Chinese gallnut was generated and gallotannins were highly accumulated, the expression levels of all three genes were significantly lower (Figure 6A). In addition, during the early developmental stages of the gallnut, *RcUGD1* and *RcUGD3* were initially downregulated and then recovered, while *RcUGD2* showed a reversed change (Figure 6B). Considering that the biosynthesis of gallotannins was consistent with the developmental process of the gallnut [19], downregulation of *RcUGD1* and *RcUGD3* guaranteed that more UDP-Glc entered gallotannin biosynthesis rather than undergoing degradation into UDP-GlcA.

### 3.8. RcUGD3 Catalyzed the Conversion of UDP-Glc to UDP-GlcA

**Figure 7:**
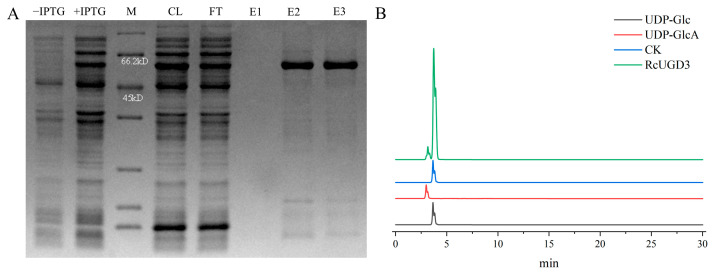
Purification and enzyme activity assay of RcUGD3 protein. (**A**) Purification of RcUGD3 protein. −IPTG indicates total protein from bacteria strain before IPTG induction; +IPTG represents total protein from bacteria strain after IPTG induction for 12 h; M stands for protein marker, CL for cell lysate, FT for flow-through fraction; and E1–E3 refer to elution fractions 1–3. (**B**) Enzyme activity assay of RcUGD3 protein. Using UDP-Glc and NAD^+^ as substrates, the purified protein was added to the enzymatic reaction. The product was detected by HPLC at a wavelength of 280 nm. UDP-Glc is the substrate standard; UDP-GlcA is the product standard; CK denotes the negative control; and RcUGD3 refers to the experimental group with purified RcUGD3 protein added to the UDP-Glc substrate for enzyme activity detection.

To figure out the enzymatic activity of RcUGD proteins, we constructed prokaryotic expression pET28a vectors for three proteins and expressed them in the *E. coli* BL21 (DE3) strain. However, only the recombinant RcUGD3 protein was successfully purified (Figure 7A). RcUGD1 and RcUGD2 were not obtained although we optimized the inducing conditions. To investigate whether the recombinant RcUGD3 protein had in vitro enzymatic activity, a reaction system was constructed using UDP-Glc as the substrate, and the catalytic products were detected by HPLC. We found that an additional peak with similar retention time to the standard UDP-GlcA was detected when the recombinant RcUGD3 protein was added to the reaction system (Figure 7B). On the other hand, in the reaction mixture without RcUGD3 protein, only a substrate peak with similar retention time to the standard UDP-Glc was found (Figure 7B). Thus, our results indicate that the recombinant RcUGD3 protein exhibited in vitro enzymatic activity to degrade UDP-Glc into UDP-GlcA.

## 4. Discussion

In plants, the same substrate being “competed for” by different enzymes is a core mechanism for regulating metabolic fluxes [37,38]. Plants use this metabolic competition to balance growth, defense, and reproduction. For instance, in *A. thaliana*, chorismate can either be converted into phenylalanine for protein and lignin syntheses or be reduced by the CRUE enzyme and subsequently transformed to produce a novel class of dihydrochorismate derivatives that act as a powerful chemical defense weapon in roots [39]. This operates more like a “diversion” mechanism. When the plant directs chorismate into the defense pathway, it reduces the flux available for phenylalanine (growth). In *Zea mays*, similar metabolic competition also lies in glycolysis and the pentose phosphate pathway, which both share the same substrate, glucose-6-phosphate, to balance energy consumption and biosynthesis of ribose and NADPH [40]. Here, in *R. chinensis*, a certain competition for UDP-Glc was also revealed. While the role of UDP-Glc metabolism in cell wall synthesis and function is well documented [41], the link between the level of UDP-Glc and the biosynthesis of hydrolyzable tannins in plants remains largely unexplored. Thus, the metabolic competition for UDP-Glc in *R. chinensis* provides new insights into gallotannin biosynthesis in plants.

As a key enzyme in the UDP-Glc branch metabolism, the activity of UGDs directly regulates the distribution of UDP-Glc in different metabolic pathways, playing a crucial regulatory role in the balance of the plant sugar metabolism network. In *R. chinensis*, gallotannins were highly accumulated in the Chinese gallnut [19], the biosynthesis of which required UDP-Glc [23]. Thus, there must be a certain competitive relationship between UGD-mediated degradation of UDP-Glc and UGT-mediated biosynthesis of gallotannin through competition for UDP-Glc. Indeed, gene expression pattern analysis showed that as compared with that in roots and stems, the expression level of *RcUGD* in leaves was significantly downregulated (Figure 6A). Moreover, previous study has revealed that the content of gallotannins in leaves was less than 5% of total dry weight, while, upon galling aphids’ feeding, gallotannins accumulated gradually and reached up to 70% of total dry weight [19]. Here, our data further show that, in the early stage of gallnut development (at 24 d post gallnut formation) when the gallotannins were greatly increased, the expression of functional *RcUGD3* was significantly downregulated too (Figure 6B). And similar findings were also reported in *G. max* [8]. Thus, we could demonstrate that, in the plant tissues with higher levels of gallotannins, the expression level of functional *RcUGD3* was relatively lower, suggesting a balance in UDP-Glc consumption.

Here, we isolated three *UGD* members in *R. chinensis* through whole-genome identification (Figure 1) of similar family members with other species [4,9,36]. The multiple sequence alignment and conserved motif analysis of the RcUGD family proteins (Figure 3) confirmed that the three RcUGD proteins we identified belong to UGD family. Meanwhile, identification of key domains and motifs in RcUGD proteins (Figure 3) further provided the structural prerequisite to catalyze the conversion of UDP-Glc to UDP-GlcA. These typical domains further supported the classification of RcUGD proteins and provided evidence for their catalytic activity. In addition, the high correlation between conserved motifs and functional domains was consistent with the motif distribution characteristics in *G. max* [42] and *Z. mays* [43], supporting their involvement in sugar metabolism and related secondary metabolic processes in *R. chinensis*. The differences in length and distribution of UTRs and CDSs among different genes reflected a certain degree of structural differentiation in the *RcUGD* gene family during evolution, which may provide a structural basis for gene expression regulation (such as transcription initiation and mRNA stability).

In plants, segmental duplication is one of the reasons for genome amplification [44]. Members of the *RcUGDs* are discretely distributed on chromosomes and are amplified through whole-genome duplication (WGD) or large segmental duplication events. No direct tandem duplication relationships have been found, suggesting that the *RcUGD* gene family may have diverged through evolutionary events such as chromosomal translocation, which was consistent with previous reports [36].

UGD, as a key enzyme in the sugar metabolism pathway, catalyzes the conversion of UDP-Glc to UDP-GlcA with the production of two NADH molecules [6]. Previous study has reported the catalytic activities of UGDs in other species. Although both ZmUGD and LbjUGD exhibit catalytic activity, LbjUGD produces more UDP-GlA than ZmUGD in vitro [43]. Here, we focused on the UGDs in the medicinal plants *R. chinensis* and found that the recombinant RcUGD3 protein exhibited a strong activity to convert UDP-Glc to UDP-GlcA as indicated by HPLC (Figure 7B). As revealed by protein sequence alignment, the RcUGD3 protein was highly homologous to AtUGD3 and AtUGD4 (Figure 1), which also possessed catalytic activity as previously reported [9]. Together with the findings of a negative correlation between the expression of *RcUGD3* and the biosynthesis of gallotannins in the Chinese gallnuts, these data suggest that RcUGD3 possibly regulates UDP-Glc degradation and affects gallotannin biosynthesis in *R. chinensis*.

Although enzymology research on UGDs has been extensively conducted in different species, functional analysis of *UGD* genes was only performed in some model plants, benefitting from the mature transgenic system. For instance, in *A. thaliana*, mutations in *UGD2* and *UGD3* resulted in plant developmental deficiency caused by a functional defect of cell wall [13]. In this study, analysis of gene expression profiles and protein catalytic activity of RcUGDs suggests that RcUGD3 may be associated with gallotannin biosynthesis in *R. chinensis*. However, whether they function in plants needs to be further investigated via a gene transformation assay in future work.

## 5. Conclusions

The traditional Chinese medicine gallnuts accumulate a high level of gallotannins, but the underlying accumulation mechanisms are largely unknown. In this study, we identified three *RcUGD* family genes in the tree species *R. chinensis*, and their structural features, expression patterns and protein catalytic activity were comprehensively analyzed. Gene expression profiling indicated that there is a negative correlation between the expression of *RcUGD3* and the biosynthesis of gallotannins. Furthermore, in vitro enzyme activity assay showed that RcUGD3 protein catalyzes the conversion of UDP-Glc to UDP-GlcA. Based on these data, we demonstrated that downregulation of *RcUGD3* limits the degradation of UDP-Glc and thus facilitates the biosynthesis of gallotannins in certain plant tissues. Our study provides new evidence connecting the enzyme gene *RcUGD3* with the accumulation of gallotannins and suggests a novel mechanism regulating gallotannin accumulation through RcUGD-mediated metabolic competition for UDP-Glc in *R. chinensis*.

## Figures and Tables

**Table 1:** Primers sequences for real-time fluorescent quantitative PCR.

Primer Name	Forward Primer (5′–3′)	Reverse Primer (5′–3′)
*qRcPP2A*	TCCACCGTCCGATCATCAGAAC	GCACGTTCCATTCCTCCACC
*qRcUGD1*	GCCTCTCCTTTGCTCTCTAGTCTC	ACACGGAGATATCAACAACAGCTAC
*qRcUGD2*	TATGATAACATGCGGAAACCTGC	GGCATAAGATAAAGTTTCGTGGC
*qRcUGD3*	GGATCCATGGCTGAAGGACATG	CTTGCTTTCACCTTGATGGTAACAC

**Table 2:** Physical and chemical properties of RcUGD proteins from *R. chinensis*.

Protein Name	Amino Acid Size	Molecular Weight	pI	Instability Index	Aliphatic Index	CRAVY
RcUGD1	481	53116.26	6.23	24.12	93.85	−0.034
RcUGD2	479	52979.83	6.06	23.85	93.63	−0.107
RcUGD3	480	52775.65	6.05	25.44	93.04	−0.056

**Table 3:** Secondary structure of the RcUGD family proteins in *R. chinensis*.

Protein Name	α-Helix (%)	Extended Chain (%)	Irregularly Curled (%)
RcUGD1	42.62%	15.18%	42.20%
RcUGD2	41.54%	15.24%	43.22%
RcUGD3	42.29%	15.21%	42.50%

## Data Availability

The original contributions presented in this study are included in the article. Further inquiries can be directed to the corresponding author(s).
